# Clinical course of idiopathic inflammatory myopathies in COVID-19 pandemic: a single-center experience

**DOI:** 10.2217/fvl-2021-0146

**Published:** 2022-06-15

**Authors:** Hakan Apaydin, Abdulsamet Erden, Serdar C Güven, Berkan Armağan, Özlem Karakaş, Bahar Özdemir, Bünyamin Polat, Mehmet Akif Eksin, Ahmet Omma, Orhan Kucuksahin

**Affiliations:** ^1^Department of Internal Medicine, Division of Rheumatology, Ankara City Hospital, Ankara, 06800, Turkey; ^2^Department of Internal Medicine, Division of Rheumatology, Ankara City Hospital, Yıldırım Beyazıt University, Ankara, 06800, Turkey

**Keywords:** corticosteroids, hydroxychloroquine, idiopathic inflammatory myopathies, immunosuppression, myositis, SARS-CoV-2

## Abstract

**Aim:** To evaluate the clinical course of idiopathic inflammatory myopathy (IIM) patients in COVID-19 pandemic, and to assess the COVID-19 outcomes in infected IIM patients. **Materials & methods:** In this study, 39 patients were evaluated retrospectively. Myositis disease activity, myositis damage index, depression, fatigue, active medical treatment, drug compliance and SARS-CoV-2 PCR test results in COVID-19 pandemic were collected. **Results:** Fourteen of these patients (35%) were detected to have a positive SARS-CoV-2 PCR test. The demographic and clinical characteristics, active medical treatment, disease activity, depression and fatigue of the patients who had undergone or not SARS-CoV-2 were similar. **Conclusion:** Our results have shown that although prevalence of COVID-19 seems to be increased in IIM patients under immunosuppressive treatment, hospitalization rates were lower and no intensive care unit admissions or deaths were observed.

COVID-19 is a respiratory infection with symptoms ranging from a mild viral illness to severe acute respiratory syndrome with organ failure and death. Most cases cause asymptomatic or mild illness, however, a significant number of patients suffer from respiratory distress requiring hospitalization and eventually a cytokine storm syndrome may occur leading to multiple organ insufficiency and death [1].

Idiopathic inflammatory myopathies (IIM) are a diverse set of acquired immune-mediated diseases that affect about 1 in 100,000 people each year [2]. Based on a cluster of some certain clinical, histological, immunopathological features and presence of associated autoantibodies, these disorders can be classified into subgroups: polymyositis (PM), dermatomyositis (DM), juvenile dermatomyositis (JDM), amyopathic dermatomyositis (ADM), inclusion-body-myositis (IBM), anti-synthetase syndrome (ASS) and immune-mediated necrotizing myopathy (IMNM) [3]. Similar to various other chronic autoimmune inflammatory rheumatic diseases, idiopathic inflammatory myopathies require immunosuppressive therapy.

COVID-19 infection causes an important problem for immunosuppressive patients with inflammatory autoimmune systemic diseases (ASDs). ASD patients have a compromised immune system and become more pronounced with immunosuppressive drugs and, they are more susceptible to viral and bacterial infections [4–7]. In an observational multicenter study, a higher prevalence of COVID-19 was found in patients with ASDs [8]. The use of immunosuppressants, involvement of major organ systems due to the disease, and associated respiratory or cardiovascular comorbidities may all be contributory.

One of the most serious complications of COVID-19 is cytokine storm syndrome which is an excessive response of the immune system to the virus [9]. Many anti-rheumatic drugs are used in cytokine storm syndrome and hyperinflammation. A genetic-based study showed that glucocorticoids, some biologic and conventional disease-modifying anti-rheumatic drugs (DMARDs: tocilizumab, hydroxychloroquine, tofacitinib, methotrexate, azathioprine, etc.) can suppress the cytokine profile in patients with rheumatoid arthritis who have severe COVID-19 [6]. Therefore, immune-compromised state caused by immunosuppressive therapies (except steroids, sulphasalazine and rituximab) in ASD patients may protect them from the major complications of COVID-19 [6,9,10].

The COVID-19 epidemic caused interruptions in the routine follow-up and treatment of patients with inflammatory rheumatic diseases like most chronic diseases. Therefore, optimal evaluation and management of these patients may have been disrupted. Furthermore, viruses have the potential to trigger immune inflammatory myopathy [3]. More than 10% of COVID-19-infected patients were reported to acquire myopathic symptoms such as myalgia, weakness and increased CK during the current epidemic [3]. A retrospective study evaluating the neurological manifestations of COVID-19 showed that infectious myositis may be a complication of SARS-CoV-2 [11]. COVID-19 may have been affected disease activity in patients with IIM. The direct or indirect impact of SARS-CoV-2 on disease activity in patients with IIM is unknown.

There are limited data regarding the outcomes of COVID-19 in IIM patients and the course of IIM in means of therapy discontinuation and flares during the pandemic. Herein, we aimed to evaluate the clinical course of IIM patients during COVID-19 pandemic with parameters such as disease and damage activity, depression, fatigue and drug compliance. In addition, we also investigated COVID-19 outcomes in infected IIM patients.

## Method

### Study design

The Ministry of Health granted official authorization to conduct this study on 31 January 2021.

### Patients

**Figure 1.**
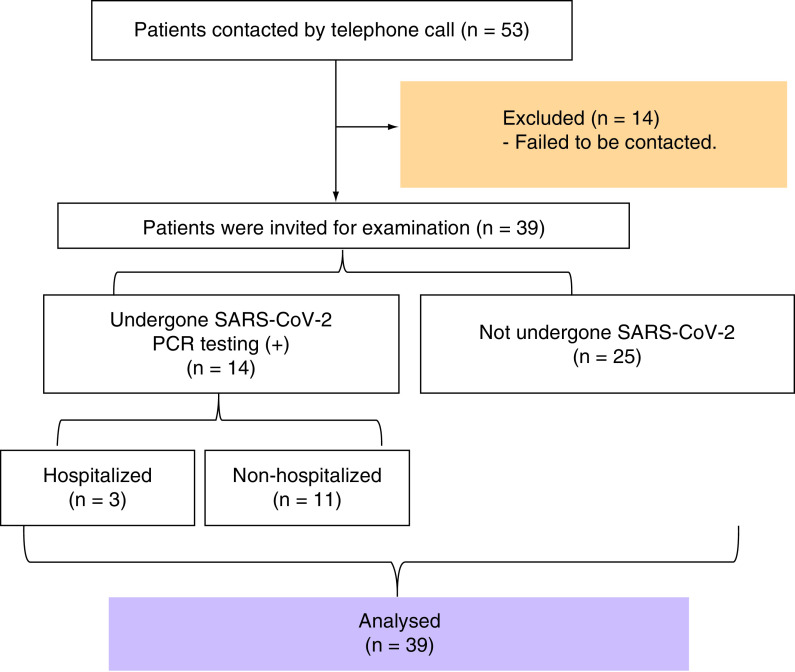
Flow chart of the study.

Between 1 January 2005 and 1 February 2021, all adult patients who have formerly diagnosed and followed with IIM by authors were retrospectively screened. Out of initial 53 subjects meeting 2017 EULAR/ACR Classification Criteria for Adult and Juvenile IIM [12], 14 observed to be lost to follow-up before the pandemic, and 39 patients were observed to keep follow-ups. All of these 39 were reached via phone numbers recorded in hospital medical records and appointed for face-to-face evaluation. The shortest disease duration was 12 months, and all patients were diagnosed before the COVID-19 pandemic. Patients under 18 years and patients diagnosed during the COVID-19 period were not included in the study. The 14 excluded patients could not be contacted or their routine examinations were interrupted (Figure 1).

### Main outcomes

In face-to-face evaluations patients were interviewed regarding demographics, history for IIM, disease activity and comorbidities, ongoing medical treatments.

Disease activity was evaluated with the myositis disease activity (MDA) assessment tool, which is a combination of the Myositis Intention-to-Treat Activity Index (MITAX) and myositis disease activity assessment visual equivalent scale (MYOACT). Over the last four weeks, the MDA evaluation tool tests potentially reversible disease behavior in muscle and six extramuscular organ systems (domains) due to the myositis disease mechanism. MYOACT extramuscular (range: 0–60) and total (range: 0–70) scores, as well as MITAX extramuscular (range: 0–54) and total (range: 0–63), were all assessed, with higher scores suggesting more disease activity [13,14].

The myositis damage index (MDI), which consists of 11 scales (cutaneous, muscular, skeletal, pulmonary, cardiovascular, peripheral vascular, endocrine, gastrointestinal, ocular organ systems, infection and malignancy), each with 2–8 items assessed as present or absent, as well as a 100-mm visual analog scale (VAS) score for each organ system, anchored at the ends and mid point, to capture severity of damage in each organ system, was used to assess IIM-related damage. Each damage item has been established, and each item must be observed for ≥6 months in order to be assessed as present and consistent with damage. A total MDI extent of damage score (0–38 in adults) was calculated by adding the presence or absence of each item. Total MDI severity (potential range: 0–110) was calculated by adding the VAS values of the 11 organ systems [15]. MDA and MDI were calculated in the post-COVID-19 period at the face-to-face evaluation.

The lassitude was evaluated with VAS score. The FACIT-fatigue (functional assessment of chronic illness therapy – fatigue) scale is a 13-item questionnaire that assesses fatigue and its effects on daily activities and functioning in a variety of chronic conditions. Tiredness, fatigue, listlessness and a lack of energy, as well as the effect of these emotions on daily functioning are all included in the instrument [16,17].

Drug compliance was assessed using visual analogue scale (VAS). Patients were given a continuous line anchored by 0 and 100% with 10% intervals and asked to label the line with their best guess about their compliance since their previous appointment using the VAS tool. Patients with drug compliance over 80% were evaluated as regular users.

The Beck Depression Inventory (BDI), a 21-item self-evaluation scale that quantitatively assesses a patient's perceived depressive symptoms, was used to evaluate IIM related mood disorders in patients. It has been proposed that those with a BDI score of 17 or higher could be considered at risk. The BDI-Turkish version's Cronbach's alpha value was 80 [18].

Patient and Physician Global Assessment of Disease Activity are basic patient-completed or physician-completed VAS, respectively, measuring the total way disease influences the patient at a point in time [19].

### Secondary outcomes

In addition to IIM-related domains, patients were asked for history of COVID-19 infection, confirmed by a positive SARS-CoV-2 PCR test and the presence of SARS-CoV-2 specific IgG antibodies enrolled in hospital records if reported by the patient. Patients of this cohort were retrospectively investigated for a SARS-CoV-2 real-time RT-PCR test and SARS-CoV-2 specific IgG antibodies results from hospital records between 11 March 2020 and 30 April 2021. All cases with an RT-PCR test were registered during the pandemic in Turkey. Anti-SARS-CoV-2 IgG antibodies were screened and registered. PCR-positive patients were asked for presence of hospitalization, intensive care unit (ICU) admission and intubation during disease course.

### Statistical analysis

The Statistical Package for the Social Sciences (SPSS) version 22 was used to conduct the statistical analyses (SPSS Inc., IL, USA). Normality of variables were investigated by visual (histogram and probability graphics) and analytical methods (Kolmogorov–Smirnov test). According to the normality of the data, descriptive statistics are presented as median and interquartile range (IQR) or mean standard deviation. Categorical variables were presented with number and percentages. For comparison of continuous variables according to normality, the Mann–Whitney-U test or the Student's t-test was utilized. The Pearson's χ^2^ test and Fisher's final test were used to evaluate categorical variables. The p-values <0.05 were considered statistically significant.

## Results

There were a total of 39 IIM patients in our cohort. Most patients were female (76.9%) and median age was 50 years. The diagnoses were DM (43%), PM (25%), ADM (12%), ASS (12%) and JDM (5%). Twenty-two (56.4%) patients were under conventional immunosuppressive treatment (azathioprine, cyclophosphamide, methotrexate and mycophenolate mofetil), 7 (17.9%) under intravenous immunoglobulin (IVIg), 12 (30.8%) under hydroxychloroquine (HCQ), 24 (61.5%) under steroids with prednisolone equivalent dose >7.5 mg/day in 7 (17.9%) and 3 (7.6%) under rituximab. While all of the patients who were under corticosteroids and IVIG continued their treatment, two of the patients receiving any other immunosuppressive treatment discontinued their treatment. Treatment was continued in 3 out of 4 rituximab recipients. A patient with polymyositis who had COVID-19 and developed pneumonia relapsed after discontinuation of azathioprine.

At least one comorbid disease was present in 51.3% of the patients. Comorbidities were hypertension in 12 patients (30.8%), type 2 diabetes mellitus in 6 (15.4%), obesity in 5 patients (12.8%), asthma or chronic obstructive pulmonary disease (COPD) in 5 patients (12.8%). The demographic and clinical characteristics of patients with IIM were given in Table 1.

**Table 1.** The demographic and clinical characteristics of patients with idiopathic inflammatory myopathy.

Characteristics	n = 39
Female: male, n (%)	30: 9 (76.9: 23.1)
Age (years), median (range)	50 (29–62)
Age at diagnosis (years), median (range)	42 (25–59)
Duration of disease (months), median (range)	60 (12–216)

Diagnosis, n (%)	
JDM DM ADM PM ASS	2 (5.1) 17 (43.6) 5 (12.8) 10 (25.6) 5 (12.8)

Disease activity at last visit, n (%)	
Remission Low disease activity Flare Active	24 (61.5) 12 (30.8) 1 (2.6) 2 (5.1)

Active medical treatments, n (%)	
Any immunosuppression IVIg Hydroxychloroquine Biological therapy Steroid use Prednisolone dose >7.5 mg/day	22 (56.4) 7 (17.9) 12 (30.8) 3 (7.6) 24 (61.5) 7 (17.9)
Patients with ≥1 comorbidities, n (%)	20 (51.3)
COVID-19 PCR positivity	14 (35.8)
Anti-SARS CoV-2 IgG antibody positivity	8 (20.5)

ADM: Amyopathic dermatomyositis; ASS: Anti-synthetase syndrome; DM: Dermatomyositis; IVIG: Intravenous immunoglobulin; JDM: Juvenile dermatomyositis; PM: Polymyositis.

Fourteen patients (35.8%) were positive for SARS-CoV-2 PCR testing, neutralizing IgG antibodies to SARS-CoV-2 were tested in a total of 18 patients and anti-SARS-CoV-2 IgG antibodies were found to be present in 8 patients (20.5%). Remission (9 [64.3] versus 15 [60]) and low disease activity (4 [28.6] versus 18 [32]) were comparable between COVID-19-positive and -negative individuals (p = 0.397; Table 2).

**Table 2.** Demographic characteristics of COVID-19-positive and -negative patients, comorbidities, duration of the disease, areas of involvement and drugs used in idiopathic inflammatory myopathy treatment.

Characteristics	COVID- 19 positive myositis (n = 14)	COVID- 19 negative myositis (n = 25)	p-value
Female sex, n (%)	9 (64.3)	21 (84.0)	0.161
Median age, years (IQR)	45 (23.25)	50 (20)	0.124
Age at diagnosis, median years (IQR)	40 (23)	42 (25)	0.224
Duration of disease, median months (IQR)	63 (90)	32 (100.50)	0.895

Diagnosis, n (%)			
JDM DM ADM PM ASS	1 (7.1) 7 (50) 1 (7.1) 4 (28.6) 1 (7.1)	1 (4) 10 (40) 4 (16) 6 (24) 4 (16)	0.818

Disease activity, n (%)			
Remission Low disease activity Flare Active	9 (64.3) 4 (28.6) 1 (7.1) 0 (0)	15 (60) 8 (32) 0 (0) 2 (8)	0.397
Patients with ≥1 comorbidities, n (%)	5 (35.7)	15 (60)	0.146
Patient relapsed in the COVID-19 pandemic, n (%)	1 (7.1)	0 (0)	0.12
The patient whose routine examination was disrupted in the COVID-19 pandemic, n (%)	7 (50)	12 (48)	0.905
The patient who regularly uses his drugs in the COVID-19 process, n (%)	11 (78.6)	22 (88)	0.434
Use of immunosuppressive, n (%) Use of IVIG, n (%) Use of hydroxychloroquine, n (%) Use of biological therapy, n (%) Steroid use, n (%) Steroid dose over 7.5 mg/day, n (%)	10 (71.4) 1 (7.1) 4 (28.6) 0 (0) 6 (42.9) 1 (7.1)	12 (48) 6 (24) 8 (32) 3 (12) 18 (72) 6 (24)	0.157 0.188 0.824 0.54 0.073 0.386
Beck depression scale, median (IQR)	15 (19.25)	13.5 (8.5)	0.354
PtGA, median (IQR)	30 (26.25)	30 (8.75)	0.852
PGA, median (IQR)	25 (16.25)	22.5 (10)	0.666
MYOACT, median (IQR)	11 (10.75)	9.5 (6.75)	0.648
MITAX, median (IQR)	2.5 (4)	3 (3)	0.469
MDI extent of damage score, median (IQR)	0.10 (0.14)	0.15 (0.07)	0.345
MDI extent of severity score, median (IQR)	0.08 (0.09)	0.10 (0.08)	0.133
VAS lassitude, median (IQR)	30 (42.5)	32.5 (15)	0.831
FACIT, median (IQR)	34 (23.25)	29.33 (13.75)	0.44

ADM: Amyopathic dermatomyositis; ASS: Anti-synthetase syndrome; DM: Dermatomyositis; FACIT: Functional assessment of chronic illness therapy – fatigue scale; IVIG: Intravenous immunoglobulin; JDM: Juvenile dermatomyositis; MDI: Myositis disease activity; MITAX: Myositis intention-to-treat activity index; MYOACT: Myositis disease activity assessment visual equivalent scale; PM: Polymyositis; PtGA: Patient global assessment; VAS: Visual analog scale.

While only one of COVID-19-positive patients relapsed in the COVID-19 outbreak, no relapse was observed in patients with COVID-19 negative. Number of patients with interrupted follow-up (7 [50] versus 12 [48]) and number of patients who continued medical treatment for IIM (11 [78.6] versus 22 [88]) were similar in both groups (p = 0.905 and p = 0.434, respectively).

The use of any immunosuppressive drug was higher in the COVID-19-positive patients than in the COVID-19-negative patients (10 [71.4]) versus 12 [48]; p = 0.157), and the use of HCQ (4 [28.6] versus 8 [32]; p = 0.824) and corticosteroids was higher in the COVID-19-negative group (6 [42.9] versus 18 [72]; p = 0.073), but no statistical significance was observed. While the use of biological therapy was absent in COVID-19-positive patients, rituximab was used in 3 COVID-19-negative patients. The disease activity and damage scores, Beck depression scores, patient and physician global assessment and fatigue scores were similar in the COVID-19-positive and -negative groups (Table 2).

Hospitalization of COVID-19-positive patients occurred in three patients (21.4%). No admission to ICU or death was observed in 14 patients with confirmed diagnosis of COVID-19. Median (min–max) length of hospital stay in COVID-19 course was 10 (7–16) days. Hospitalized patients had a higher median(min–max) age than non-hospitalized patients (57 [51–61] versus 37 [24–60]). Hospitalized patients had longer disease duration and more comorbidities. While the use of immunosuppressive therapy was similar in hospitalized and non-hospitalized patients (8 [72.7] versus 2 [66.7]), hydroxychloroquine use was higher than in non-hospitalized patients (4 [36.4] versus 0 [0]) (Table 3). Active medical treatment agents and COVID-19 outcomes were given in Table 3.

**Table 3.** Active medical treatment, COVID-19 course and post-COVID-19 course related to hospitalization in idiopathic inflammatory myopathy patients with positive SARS-CoV-2.

Characteristics	Myositis patients with positive SARS-CoV-2	
	Non-hospitalized (n = 11)	Hospitalized (n = 3)
Age, years, median (min-max)	37 (24–60)	57 (51–61)
Gender, female, n (%)	8 (61.5)	3 (37.5)
Duration of disease, median (range)	60 (7–240)	128 (72–156)
Patients with ≥1 comorbidities, n (%)	2 (18.2)	3 (100)

Comorbidities, n (%)		
Hypertension	2 (18.2)	2 (66.7)
Diabetes	1 (9.1)	1 (33.3)
Obesity (BMI>30)	0 (0)	3 (100)
Asthma or COPD	0 (0)	3 (100)

Active medical treatment for myositis, n (%)		
Hydroxychloroquine	4 (36.4)	0 (0)
Immunosuppressive treatment	8 (72.7)	2 (66.7)
Steroid	5 (45.5)	1 (33.3)
Steroid dose 7.5 mg/day or more	1 (9.1)	0 (0)
IVIg	1 (9.1)	0 (0)

COVID-19 course		
Length of hospital stay (days), median (range)	–	10 (7–16)
ICU admission, n (%)	–	0 (0)
Death, n (%)	–	0 (0)

Post-COVID-19 course		
Beck depression scale, median (range)	15 (2–28)	24 (4–30)
PtGA, median (range)	30 (10–50)	45 (25–70)
PGA, median (range)	20 (15–40)	35 (35–50)
MYOACT score, median (range)	10 (3–18)	18 (15–18)
MITAX score, median (range)	2 (1–5)	6 (5–7)
MDI extent of damage score, median (range)	0.10 (0.03-0.31)	0.10 (0.07-0.26)
MDI extent of severity score, median (range)	0.07 (0.02-0.15)	0.10 (0.09-0.13)
VAS lassitude, median (range)	30 (5–70)	75 (0–80)
FACIT, median (range)	34 (18–50)	10 (6–48)

FACIT: Functional Assessment of Chronic Illness Therapy - Fatigue scale; ICU: Intensive care unit; IVIg: Intravenous immunoglobulin; MDI: Myositis disease activity; MITAX: Myositis intention-to-treat activity index; MYOACT: Myositis disease activity assessment visual equivalent scale; PtGA: Patient global assessment; VAS: Visual analog scale.

When pre-COVID-19 and post-COVID-19 laboratory parameters were compared in IIM patients with COVID-19, no statistical difference was found, including creatinine kinase (CK) (81.5 [77–262] versus 80 [69–150]; p = 0.638) (Table 4). A patient with polymyositis who developed mild COVID-19 pneumonia was hospitalized and developed a relapse. This patient's CK value was increased from 488 µ/l to 2813 µ/l in the post-COVID-19 period. In accordance with CK elevation, significant muscle weakness developed in the post-COVID-19 period.

**Table 4.** Comparison of pre- and post-COVID-19 laboratory parameters in IIM patients with COVID-19.

	Pre-COVID-19, median (IQR)	Post-COVID-19, median (IQR)	p-value^†^
WBC (per mm^3^)	7875 (7090- 9540)	7490 (6300–9680)	0.187
Neutrophil (per mm3)	5315 (3490–7110)	4840 (3600–7250)	0.258
Lymphocyte (per mm^3^)	1740 (1450–2120)	2020 (1330–2200)	0.875
AST (µ/l)	26 (20–55)	21.5 (18–50)	0.582
ALT (µ/l)	37 (22–55)	31.5 (22–48)	0.814
LDH (µ/l)	229 (224–297)	265 (247–292)	0.875
Creatinine (mg/dl)	0.7 (0.58–0.77)	0.7 (0.66–0.77)	0.346
CK (µ/l)	81.5 (77–262)	80 (69–150)	0.638
ESR (mm/h)	20.5 (10–32)	17.5 (8–29)	0.388
CRP (mg/l)	3.5 (3–12)	3 (2–6)	0.959

All laboratory parameters have been calculated as median (IQR).

† p < 0.05. Wilcoxon signed ranks test.

ALT: Serum alanine aminotransferase; AST: Serum aspartate aminotransferase; CK: Creatinine kinase; CRP: C-reactive protein; ESR: Erythrocyte sedimentation rate; IQR: Interquartile range; LDH: Lactate dehydrogenase; WBC: White blood cell count.

Twelve IIM patients (30.7%) had interstitial lung disease (ILD) in our cohort. Five of these patients were ASS (45.7%), 3 were DM (25 %), two were PM (%16.7) and ADM (%16.7). Positive SARS-CoV-2 was detected in 4 (33.3%) patients, three of them were symptomatic (25%). COVID-19 pneumonia developed in only one patient and this patient was hospitalized. Pulmonary involvement was mild in this patient, he received favipiravir treatment for COVID-19, methylprednisolone 4 mg/day for IIM was continued while azathioprine was discontinued during COVID-19 infection. ICU admission and death was not seen in this patient. Clinical characteristics of IIM patients with interstitial lung disease involvement were given in Table 5.

**Table 5.** Clinical characteristics of idiopathic inflammatory myopathy patients with interstitial lung disease involvement.

Characteristics	Patients with interstitial lung disease (n = 12)
Female sex, n (%)	9 (75)
Median age, (IQR) years	44 (29)
Age at diagnosis, median (IQR) years	40 (20.5)
Duration of disease, median (IQR) months	60 (111)

Diagnosis, n (%)	
JDM DM ADM PM ASS	0 (0) 3 (25) 2 (16.7) 2 (16.7) 5 (41.7)

Disease activity, n (%)	
Remission Low disease activity Flare Active	6 (50) 5 (41.7) 1 (8.3) 0 (0)
Patients with ≥1 comorbidities, n (%)	6 (50)
Patient relapsed in the COVID-19 pandemic, n (%)	1 (8.3)
Use of immunosuppressive, n (%) Use of IVIG, n (%) Use of hydroxychloroquine, n (%) Use of biological therapy, n (%) Steroid use, n (%) Steroid dose over 7.5 mg/day, n (%)	10 (83.3) 2 (16.7) 2 (16.7) 1 (8.3) 9 (75) 3 (25)
Positive SARS-CoV-2	4 (33.3)
Symptomatic COVID-19	3 (25)
Hospitalization	1 (8.3)
COVID-19 pneumonia	1 (8.3)

COVID-19 course	
ICU admission, n (%) Death, n (%)	0 (0) 0 (0)

ADM: Amyopathic dermatomyositis; ASS: Anti-synthetase syndrome; DM: Dermatomyositis; ICU: Intensive care unit; IVIG: Intravenous immunoglobulin; JDM: Juvenile dermatomyositis; PM: Polymyositis.

## Discussion

The present study investigated the clinical course of IIM in COVID-19 pandemic. The disease activity and damage scores, Beck depression scores, patient and physician global assessment and fatigue scores were similar in the COVID-19-positive and -negative groups. Continuation of therapy was overall high in our cohort, and one COVID-19-positive patients relapsed but no relapse was observed in patients with COVID-19-negative group in the COVID-19 pandemic. Our results demonstrated that 14 (35.8%) of IIM patients had a positive SARS-CoV-2 PCR test, three of them were hospitalized and none were admitted to ICU or died. The use of immunosuppressive drugs was higher in the COVID-19-positive group compared with COVID-19-negative group, but no statistical significance was observed. None of the HCQ, IVIg and prednisolone equivalent dose >7.5 mg/day users were hospitalized. Age, disease duration and rate of comorbidities were found to be higher in hospitalized patients.

According to retrospective cohort studies, risk factors for COVID-19-associated poor outcome include immunocompromised status, advanced age and comorbidities such as chronic renal disease, diabetes mellitus, hypertension, cardiovascular disease, chronic lung disease and obesity [20,21]. These diseases frequently accompany patients with rheumatic diseases [22]. In a multicenter cohort study conducted in Spain the clinical outcomes of hospitalized patients with COVID-19 and chronic inflammatory ASDs were investigated [23]. Independent factors associated with severe COVID-19 in logistic regression analysis were found to be increased age, male gender and having a connective tissue disease. In addition, studies have shown that SARS-CoV-2 infection is more severe in men, and female dominance in systemic autoimmune diseases may represent a protective feature [23]. In our cohort, female gender was more frequent as expected in autoimmune rheumatic diseases however, older age, male gender and frequency of comorbidities were associated with hospitalization.

In a study of 320 Italian patients with chronic arthritis receiving immunosuppressive therapy, four confirmed cases and four other cases highly suggestive of SARS-CoV-2 were reported, no cases developed serious complications or died [24]. Furthermore, in comparison to the general population, patients with rheumatoid arthritis treated with biological DMARDs or targeted synthetic DMARDs did not appear to have an elevated risk of life-threatening complications from SARS-CoV-2. An 80-year-old woman with PM on azathioprine was described with a benign COVID-19 prognosis in a case series of four patients with rheumatologic diseases [25]. Immunosuppressive medications were not substantially related with an increased risk of ICU hospitalization, mechanical ventilation or death in COVID-19, according to preliminary research from the Italian Registry of the Italian Society of Rheumatology [24]. In Italy, patients who had previously received a solid organ transplant and were undergoing cancer treatment, the majority of whom were on glucocorticoids, did not develop substantial COVID-19 complications [26]. Dexamethasone treatment was linked to decreased 28-day mortality in a subgroup of COVID-19 patients receiving respiratory support in a trial of hospitalized COVID-19 patients [27]. In an observational multicenter study, the prevalence of COVID-19 was investigated by a 6-week phone questionnaire of Italian patients with 1641 autoimmune systemic diseases during the COVID-19 pandemic. In comparison to the ‘Italian general population’, the complete series of ASDs demonstrated a remarkably higher proportion of individuals with a definitive diagnosis of COVID-19 disease, highly suspected COVID-19 disease, or both disorders. In addition, individuals with varied ‘connective tissue diseases’ had a considerably greater incidence of a definite + high suspicion diagnosis of COVID-19 disease compared with the ‘inflammatory arthritis group’ or patients who were not using any traditional synthetic disease-modifying anti-rheumatic medicines [8]. Our findings showed that 14 patients with confirmed diagnosis of COVID-19 had no ICU admission or death, hospitalization occurred in 3 (21.4%) patients and four patients who received HCQ, one patient who took IVIg and prednisolone equivalent dose >7.5 mg/d did not have hospitalization. While the frequency of COVID-19 in our cohort was 35%, its frequency in the general population in Turkey is reported to be 5.2% [28]. According to our results, IIM patients under immunosuppressive treatments have an increased rate of SARS-CoV-2 infection however rates of hospitalization and mortality were absent which may be related to baseline administration of immunosupressants inhibiting a hyperimmune reaction. Emerging evidence suggest that various immunosuppressants, JAK inhibitors and/or biologics could alleviate the serious results of COVID-19, favoring their use in the management of rheumatic disease during the pandemic [29]. On the contrary, rituximab has been associated with a poor prognosis in rheumatic patients with COVID-19 [30]. Rituximab, a potent inhibitor of CD20 B-cells, reduces antibody formation and thus the ability of humoral immune system to fight viral infections [31]. Immunosuppression did not adversely affect the course of COVID-19 in our patients because three hospitalized patients recovered without admission to the ICU. None of the three rituximab recipients developed COVID-19, so the effect of rituximab in IIM patients with COVID-19 could not be evaluated.

American College of Rheumatology (ACR) recommends that patients with stable rheumatic disease may continue immunosuppressive drugs and the dose of immunosuppressants may not be reduced for patients with a history of organ-threatening rheumatic disease [32]. Our data support this recommendation and our cohort reveals that immunosuppression may not be associated with adverse outcomes in IIM patients.

Beydon *et al.* [33] reported the first case of MRI-proven myositis in a patient of COVID-19 in April 2020 in France. In another study, myalgia is present in 36% of COVID-19 patients and 16–33% have elevated CK levels [4]. In a patient with immune mediated necrotizing myopathy, disease flare was observed in a single patient as a result of discontinuation of immunosuppressive drugs due to COVID-19 [34]. An observational analysis on the prevalence and clinical course of COVID-19 in rheumatic disorders revealed that approximately 60% of patients were on conventional synthetic disease modifying drugs, 25 patients on biological agents and 64.2% on corticosteroids [35]. Only 5 out of 123 patients discontinued their rheumatologic treatments and 115 were reported to maintain stable disease activity without exacerbation. Clinical outcomes (major/minor relapse) and prescription modifications were compared between IIM subgroups in a prospective cohort study of patients with IIM in India [36]. Over a quarter (26.8%) reported relapse (15.5% minor and 11.3% major). In our study, the patient who regularly used their drugs during COVID-19 pandemic (78.6% vs 88%) were similar in both COVID-19-positive and -negative groups. In the present study, one PCR-positive patient relapsed, while none of the PCR-negative patients relapsed. Patients continued their medication at a high rate in both groups.

Pulmonary and muscular manifestations are common in both COVID-19 and some autoimmune rheumatic diseases, such as IIM, and diagnosis can be difficult as they share common clinical and radiological features [37,38]. DM patients, especially ADM patients may have interstitial lung disease and COVID-19 should be differentiated from ILD attributed to connective tissue disease, particularly rapidly progressing (RP)-ILD associated with DM/ADM. COVID-19 pneumonia has a radiological appearance that is similar to ILD in anti-MDA5-positive myositis with diffuse ground glass opacities (GGOs). High-dose steroids, immunosuppressants, and anti-cytokine treatment can be used to treat both of these disorders [39]. Cao *et al.* described a case of a woman who was followed with fever, cough and rapid lung injury during the pandemic [40]. The patient had a suspected epidemiological history and chest CT scans revealed lung damage caused by COVID-19, but the anti-Ro52 antibody was strongly positive. She died a month after being diagnosed with ADM-associated RP-ILD. In our cohort, 12 patients (30.7%) had ILD, positive SARS-CoV-2 was detected in four (33.3%) patients. COVID-19 pneumonia developed in one patient and ICU admission and death was not seen in this patient. During the COVID-19 outbreak, it is crucial to cautiously evaluate patients with IIM-associated ILD.

The changes in the psychological state of the patients with rheumatic diseases during the COVID-19 outbreak were evaluated with a web-based cross-sectional study in Turkey [41]. The psychiatric status of the patients was evaluated using the hospital anxiety and depression scale. The level of their psychological distress were found to be similar to those of the healthy group (teachers/academic staff). The prevalence of anxiety and depression in patients with rheumatoid arthritis (RA) before and during the COVID-19 pandemic was researched [42]. While the anxiety level in RA patients was found to be higher than the pre-pandemic levels, the depression levels in several patients with RA did not change during the pandemic, and it was reported that the number of patients reporting suspected depression increased remarkably after the COVID-19 outbreak. We found no statistically significant difference in depression score between COVID-19-positive and -negative patients in our cohort in IIM patients, but we found more severe depression in hospitalized patients than non-hospitalized patients in the post-COVID-19 course.

The retrospective structure of the study and the small sample size were major limitations, but IIM patients are rare diseases, hence the number available is valuable. Another limitation of our study is that disease activity and damage parameters may not be fully reflected because they were not evaluated immediately after contact, and, due to its retrospective design and data extraction from medical records, some clinical information may have been underreported.

## Conclusion

All in all, despite the fact that 35.8% of our IIM patients suffered from COVID-19, no COVID-19-related deaths or ICU admissions were observed, even in our patients with interstitial lung disease. Our results did not suggest any negative effect of ongoing immunosuppressive treatment on COVID-19 outcomes. Findings of this study did not imply a worse COVID-19 prognosis in IIM patients under immunosuppressive treatment, however, larger studies are needed for more definite conclusions.

**Summary points**

- COVID-19 infection causes an important problem for immunosuppressive patients with inflammatory autoimmune systemic diseases (ASDs).
- In this study, we aimed to evaluate the clinical course of idiopathic inflammatory myopathy (IIM) patients during COVID-19 pandemic with parameters such as disease and damage activity, depression, fatigue and drug compliance. In addition, we also investigated COVID-19 outcomes in infected IIM patients.
- There was a total of 39 IIM patients in our cohort. Fourteen patients (35.8%) had positive SARS-CoV-2 PCR test and were diagnosed with COVID-19. Remission and low disease activity were comparable between COVID-19-positive and -negative individuals.
- While only one of COVID-19-positive patients relapsed in the COVID-19 outbreak, no relapse was observed in patients with COVID-19 negative.
- The disease activity and damage scores, Beck depression scores, patient and physician global assessment and fatigue scores were similar in the COVID-19-positive and -negative groups.
- Hospitalization of COVID-19-positive patients occurred in 3 patients (21.4%). No admission to ICU or death was observed in 14 patients with confirmed diagnosis of COVID-19. Hospitalized patients had a higher median age than non-hospitalized patients. Hospitalized patients had longer disease duration and more comorbidities.
- Our results have shown that although prevalence of COVID-19 seem to be increased in IIM patients under immunosuppressive treatment, hospitalization rates were lower and no intensive care unit admissions or deaths were observed.
